# A braided cancer river connects tumor heterogeneity and precision medicine

**DOI:** 10.1186/s40169-016-0123-4

**Published:** 2016-10-20

**Authors:** James J. Hsieh, Emily H. Cheng

**Affiliations:** 1 Department of Medicine, Memorial Sloan Kettering Cancer Center, New York, NY 10065 USA; 2 Department of Pathology, Memorial Sloan Kettering Cancer Center, New York, NY 10065 USA; 3 Human Oncology and Pathogenesis Program, Memorial Sloan Kettering Cancer Center, New York, NY 10065 USA

**Keywords:** Tumor heterogeneity, Precision medicine, Braided cancer river model, Kidney cancer, MTOR inhibitors, VEGF inhibitors, Branched evolution, Parallel convergent gene/pathway/capability/function evolution

## Abstract

With the ever-increasing complexity of tumor heterogeneity (TH) discovered through cancer genome sequencing, it is apparent that TH has become the biggest hurdle for precision cancer therapeutics. Through studying the genomics of exceptional responders to targeted therapeutic agents in kidney cancer, we demonstrated parallel convergent gene/pathway/capability/function evolution of kidney cancer in the context of TH, which prompted us to propose a new cancer evolution model “the braided cancer river model”. Based on this model, we might be able to outsmart a given cancer type within an individual patient through simultaneously inhibiting preferred parallel pathways or sequential nodes. Thus, the goals of this perspective are to define tumor heterogeneity, discuss tumor evolution, introduce braided cancer river model, and improve precision medicine.

Improving precision has always been a common goal of the human race, which in turn drives the continuous progresses of all disciplines. Cancer has been a mystery in health concerning the origin, the identity, the manifestation, the management, and the outcome since its existence. With the advent of modern molecular, imaging, and treatment technologies in the latter half of the 20th century, we began to understand the complexity of cancer and are now gearing towards delivering precision therapeutics for personalized cancer care in the 21st century. Currently, we have surgery and radiation for localized cancer control, and chemotherapeutic, targeted, and immunotherapeutic agents for systemic management of advanced cancers. With the ever-increasing treatment options, we are now in an era of precision cancer medicine that promises maximum specificity and effectiveness. To discuss how we might improve specificity in precision medicine, we will concentrate on issues concerning tumor heterogeneity. We can categorize tumor heterogeneity into three levels, i.e. macroscopic, microscopic, and molecular. Macroscopically, factors involved are individual cancer patient’s race, gender, age, and the organ/tissue origin of contracted cancer; microscopically, factors are histology and tumor microenvironment; and molecularly, factors are genetics and epigenetics [1]. Beside these foreseeable layers of complex, several contemporary studies opened our eyes to the unprecedented scale of intra-tumor heterogeneity (ITH) and inter-metastases heterogeneity (IMH) within an individual patient of a given cancer. Facing this daunting tumor heterogeneity, curing cancer with precision therapeutics seems infeasible. Yet, we are making significant progress in converting metastatic cancer from an acute fetal disease to a manageable chronic illness, and even in some scenarios provide continuous remission without ongoing treatment, e.g. kidney cancer [1].

The “natural selection” theory by Charles Darwin stipulated that all organisms arise and develop through small, inherited variations that increase the individual’s ability to compete, survive, and reproduce. Cancer is a disease of the genome. Accordingly, the creation, the increased proliferation, the uncontrolled migration, and the ultimate demise of individual cancer cells follow Darwinian principles. Cancer cell originates from the same copy of DNA as its host and acquires new capability through mutations. The drastic, uncontrollable, rapid evolution of cancer cell ends with the demise not only of the host but also the renegade cancer cells that are constantly in chaos.

Central to the cancer evolution principle is the ability of cancer cells to rapidly diversify and compete as “clonal evolution of tumor cells” [2], favoring linear evolution in creating the fittest clone [3]. With the advent of next generation sequencing and the insight of tumor heterogeneity, the extensive ITH was unveiled by the Gerlinger et al., where the authors examined tumor DNA of different regions of human clear cell renal cell carcinoma (ccRCC) and highlighted branched evolution [4]. Since then, divergent/branched evolution has been demonstrated as a common phenomenon in multiple cancer types. The complexity of cancer cell spreading between primary and metastatic tumors and among metastatic lesions was best illustrated by Gundem et al. [5], where the authors utilized whole genome sequencing to deduce the recurrent seeding-colonizing-spreading cycles in prostate cancer. They found metastasis-to-metastasis colonization was common; metastasis-to-metastasis spread can initiate through either de novo monoclonal seeding or simultaneous transfer of multiple tumor clones; and multiple colonizing events took place among metastases over time [5].

Both ITH and IMH thrive on diversifying through mutations and epigenetic changes, which can be viewed as an individual ever-branching cancer tree. The trunk-branch model concerning ITH and IMH pinpoints the strategy for effective cancer control through targeting “trunk” lesions [6], explaining the known success of multiple kinase inhibitors in treating certain human cancers bearing truncal activating mutations at respective kinases [7]. Another key finding in the Gerlinger et al. paper is that different mutations at the very same genes were detected in different regions of the same tumor. This observation underscores the notion that upon expansion, invasion, and metastasis tumor would do whatever is necessary to activate or inactivate “certain” genes through convergent evolution in order to increase fitness [8]. This data is consistent with the “Hallmarks of Cancer” concept proposed by Weinberg et al. [9] where certain biological capabilities are acquired during the multistep development of human tumors [10, 11]. In other words, convergence on genes, pathways, and capabilities/functions confers the ultimate phenotypes in cancer evolution.

For ccRCC, two classes of targeted agents have been approved that target VEGF and mTOR pathways [12] and collectively improved overall survival of metastatic patients [11, 13]. The median progression free survival (PFS) for metastatic ccRCC patients receiving mTORC1 inhibitors is <6 months. Nevertheless, a small group of patients experienced markedly longer survival [14, 15]. For example, a median PFS of 28 months was reported in an outlier study of five patients with metastatic ccRCC [14]. In three of five patients, multi-region analysis revealed marked ITH, whereas results revealed pathway convergence upon mTOR pathway activation [11, 14]. For example, two distinct and spatially separate mutations in *TSC1* and *MTOR,* along the PI3 K/AKT/mTOR pathway, that activate mTOR kinase through different mechanisms were detected in different regions of the same tumor [14]. What can we learn from studying targeted therapeutic outliers of a given cancer type, which gives rise to seemingly contradictory phenotypes, i.e. diversification in mutations and durable benefit on one drug? The recurrent theme is parallel/convergent evolution on select sets of oncogenic pathways [11, 14, 16]. Importantly, such pathway or phenotype convergence takes place within and among tumors, and is probably shared by a given cancer type in the presence of ITH and IMH [11]. Based on these insights on parallel/convergent gene/pathway/function (capability)/phenotype evolution [11, 14, 16], kidney cancer development may be better visualized as a braided river with the capacity to diverge and converge rather than an ever-branching tree. The riverhead is analogous to the trunk mutation, originating with a ubiquitous driver event. The heterogeneous mutations previously ascribed to the branches of tree become tributaries along the river, which retain the capability to become driver mutations and converge with other spatially or temporally distinct mutations that affect the same genes or components along critical oncogenic or tumor suppressor pathways inherent to a given cancer type [11].

This braided cancer river model illustrates parallel and convergent events occurring throughout tumorigenesis. Starting from initiating driver mutations, this model depicts the stepwise acquisition of different driver mutations (early, intermediate, late and speedy drivers) during cancer evolution. Based on this model, one can envision how we might be able to outsmart cancer before resistance to targeted agents occur through agents simultaneously inhibiting parallel pathways or sequential nodes, which could explain the therapeutic benefits of administering lenvatinib (a VEGF and FGF inhibitor) plus everolimus (an mTOR inhibitor) [17] and cabozantinib (a VEGF and c-MET inhibitor) [18] in ccRCC patients who have failed frontline anti-VEGF therapies and the combination of bevacizumab (an anti-VEGF-A antibody) and everolimus in non-clear cell RCC with papillary features [19]. Furthermore, with recent success in cancer therapy using immune checkpoint blockade (ICB) antibodies [20], one might consider combining these two different strategies, namely targeted agents and cancer immunotherapies, as the future precision cancer medicine. In fact, such approaches are being explored in phase III trials.

In conclusion, with the advent of NGS and the affordable cost for sequencing tumors, we can now evaluate tumors at base-pair resolution and with the availability of large cancer genomics dataset, we should be able to better model individual tumor evolution in real time with precision. The future of cancer therapeutic is to target cancer cells at fine resolution, fulfilling the promise of precision medicine, which will likely reduces the eventual economic and social burdens imposed by cancer as a whole.
